# Intra-Discrete Typing Unit TcV Genetic Variability of *Trypanosoma cruzi* in Chronic Chagas' Disease Bolivian Immigrant Patients in Barcelona, Spain

**DOI:** 10.3389/fcvm.2021.665624

**Published:** 2021-05-20

**Authors:** Maykon Tavares de Oliveira, Elena Sulleiro, Maria Cláudia da Silva, Aroa Silgado, Marta de Lana, João Santana da Silva, Israel Molina, J. Antônio Marin-Neto

**Affiliations:** ^1^Department of Infectious Diseases, Vall d'Hebron University Hospital, Universitat Autònoma de Barcelona, PROSICS Barcelona, Barcelona, Spain; ^2^Cardiology Division, Department of Internal Medicine, Medical School of Ribeirão Preto, University of São Paulo, São Paulo, Brazil; ^3^Department of Microbiology, Vall d'Hebron University Hospital, Universitat Autònoma de Barcelona, PROSICS Barcelona, Barcelona, Spain; ^4^Department of Biochemistry and Immunology, Ribeirão Preto Medical School, University of São Paulo, São Paulo, Brazil; ^5^Pharmacy School and Center of Research in Biological Sciences, Federal University of Ouro Preto, Ouro Preto, Brazil; ^6^Fiocruz-Bi-Institutional Translational Medicine Plataform, Ribeirão Preto, Brazil

**Keywords:** Chagas disease, *Trypanosoma cruzi*, DTU TcV, genetic variability, cardiac form

## Abstract

**Background:** *Trypanosoma cruzi* has a high rate of biological and genetic variability, and its population structure is divided into seven distinct genetic groups (TcI-TcVI and Tcbat). Due to immigration, Chagas disease (ChD), caused by *T. cruzi*, has become a serious global health problem including in Europe. Therefore, the aim of this study was to evaluate the existence of genetic variability within discrete typing unit (DTU) TcV of *T. cruzi* in Bolivian patients with chronic ChD residing in Barcelona, Spain.

**Methods:** The DNA was extracted from the peripheral blood of 27 patients infected with *T. cruzi* DTU TcV and the fragments of the genetic material were amplificated through the low stringency single primer-polymerase chain reaction (LSSP-PCR). The data generated after amplification were submitted to bioinformatics analysis.

**Results:** Of the 27 patients evaluated in the study, 8/27 (29.6%) were male and 19/27 (70.4%) female, 17/27 (62.9%) were previously classified with the indeterminate clinical form of Chagas disease and 10/27 (37.1%) with Chagas cardiomyopathy. The LSSP-PCR detected 432 band fragments from 80 to 1,500 bp. The unweighted pair-group method analysis and principal coordinated analysis data demonstrated the existence of three distinct genetic groups with moderate-high rates of intraspecific genetic variability/diversity that had shared parasite's alleles in patients with the indeterminate and cardiomyopathy forms of ChD.

**Conclusions:** This study demonstrated the existence of a moderate to high rate of intra-DTU TcV variability in *T. cruzi*. Certain alleles of the parasite were associated with the absence of clinical manifestations in patients harboring the indeterminate form of ChD. These results support the need to search for increasingly specific targets in the genome of *T. cruzi* to be correlated with its main biological properties and clinical features in patients with chronic ChD.

## Introduction

Chagas disease (ChD) is caused by the hemoflagellate protozoan *Trypanosoma cruzi* and chronic Chagas cardiomyopathy is the most severe manifestation (1). According to recent data from the World Health Organization (WHO), ~6 to 7 million people are chronically infected with *T. cruzi* worldwide, and more than 75 million individuals are at risk of infection (2).

The genetic structure of *T. cruzi* is currently divided into seven distinct genetic groups, also known as discrete typing units (DTUs), TcI–TcVI, and TcBat (3, 4). *T. cruzi* presents a high rate of biological and genetic variability (5) and these differences may be linked to the main biological parameters of the different strains of *T. cruzi*, such as geographical distribution and human clinical manifestations of ChD. However, no previous studies have presented sufficient data to confirm effectively such potential correlations (6).

The area of spread of ChD is wide across the American continent. One of the most notable changes in the epidemiology of parasitic diseases in recent decades is the emergence of ChD in European countries, and the associated risk of *T. cruzi* transmission outside endemic areas (7). Europe is currently hosting large immigrant populations, with recent data estimating that immigrant populations represented 8.7% of the total European population in 2010 (8). The prevalence of ChD infection in Latin American immigrants living in Europe is estimated in 4.2%, with the highest prevalence among migrants from Bolivia (18.1%) and Paraguay (5.5%) (9). Population movement over recent years has led to an increased prevalence of ChD in these countries, primarily due to high numbers of Latin American immigrants chronically infected with *T. cruzi* (10).

Although direct vector transmission cannot occur in Europe, ChD can be transmitted in non-endemic countries *via* blood transfusion and organs transplantation, or even vertical transmission (9). Measures to control vertical transmission have been designed and implemented in some countries to avoid spreading ChD in Europe, although these measures have not been shown to be entirely viable (11).

Assessing the real burden and implications for public health of ChD in European countries is crucial. Therefore, this study aimed to evaluate the intraspecific genetic variability of DTU TcV from *T. cruzi* in immigrant patients with chronic ChD residing in Barcelona, Spain, and to correlate the genetic differences intra-DTU with the correspondent clinical forms of ChD.

## Materials and Methods

### Patients and Blood Samples

Were evaluated twenty-seven patients with ChD infected by DTU TcV previously genotyped by our group (12). Diagnosis of ChD was confirmed for all 27 patients *via* two positive serological tests and real-time PCR, according to the WHO recommendations. All patients were clinically evaluated at Vall d'Hebron University Hospital, Barcelona, Spain, between 2015 and 2019. From all patients, 5 mL of peripheral blood were collected and mixed with an equal volume of guanidine 6 M/EDTA 0.2 M pH 8 (13). The Guanidine-EDTA Blood lysates (GEB) were boiled for 15 min, incubated at room temperature for 24 h, and stored at 4°C until use (14).

### Extraction of DNA From Blood/Guanidine and EDTA Samples

DNA was extracted from 200 μL of guanidine/EDTA blood (GEB) samples and eluted in 55 μL using the NucliSens easyMAG® system (Biomerieux, France), according to the manufacturer's instructions.

### Clinical Evaluation of Patients

The 27 patients with positive serology and real-time PCR for ChD, were clinically evaluated at the Infectious Diseases Division, Vall d'Hebron University Hospital, Barcelona, Spain, through anamnesis, 12-lead electrocardiogram, chest, esophageal, and colon X-rays, and rest transthoracic echocardiography. The patients were classified as having different clinical forms of chronic ChD (15, 16).

### Intra-DTU TcV Genetic Variability

To assess the intraspecific genetic variability of the previously genotyped *T. cruzi* DTU TcV (11) present in the peripheral blood of patients with chronic ChD, low stringency single primer (LSSP-PCR) methodology (17) was performed. To obtain the genetic signature of *T. cruzi* kDNA, the following steps were performed: (A) amplification of the 330 bp fragment specific to *T. cruzi* kDNA (18). The amplified products were run on a 1.5% agarose gel (Sigma®), stained with Syber (Midori Green Advanced DNA Strain, Nippon Genetics Europe Gmbh) and viewed on the Biorad photo documentation platform (Molecular Imager, Gel DOC XR, Imaging System). The 330 bp fragments were removed from the agarose gel, heated to 100°C, and diluted in ultrapure water at a 1:10 dilution. (B) DNA of the diluted 330 bp band fragments was subjected to a new amplification cycle using the LSSP-PCR technique with the S35G^*^ primer (5′-AAA TAA TGT ACG GGG GAG AT-3′). A volume of 1 μL of diluted DNA was added to the 10 μL of the reaction mixture containing 6.38 μL of sterile milli-Q water, 2.0 μL of sample buffer, 0.2 μL of each deoxynucleotide (dATP, dCTP, dGTP, and dTTP–Sigma, St. Louis, MO, USA), 0.1 μL of the S35G ^*^ primer (450 μM), and 0.32 μL of Taq DNA polymerase (Go Taq-Promega) (18). Amplification occurred under the following conditions: an initial DNA denaturation stage at 95°C for 5 min, annealing at 30°C for 1 min, and extension at 72°C for 1 min, followed by 40 amplification cycles consisting of a denaturation step at 94°C for 1 min, one cycle of annealing at 30°C for 1 min, followed by a final extension step at 72°C for 10 min. The LSSP-PCR products were separated in 6% polyacrylamide gel electrophoresis and revealed with NaOH and formaldehyde after silver staining (19). After photo documentation the differences in the patterns of band profiles were analyzed *via* bioinformatics tests.

### Bioinformatics Analysis

The LSSP-PCR profiles were used to build a presence/absence matrix of each visualized band, allowing a similarity analysis to be built using NTSYSpc software. The relationships between *T. cruzi* strains were estimated using a dendrogram representative of the LSSP-PCR data. These were constructed based on the coefficient of association (20) and the analysis of unweighted peer groups [unweighted pair-group method analysis (UPGMA)] using Mega 6.04 Beta software. To estimate Shannon's diversity, the cophenetic correlation coefficient, heterozygosity by locus (He), and principal coordinated analysis (PCoA) were performed using the GenAlEx 6.5 software. In this case, a genetic distance matrix was built. This calculation of genetic distances took place in pairs for binaries. Data followed the methods of Huff and collaborators (21), wherein any comparison with the same state generates a value of 0 (for both 0 vs. 0 comparisons and comparisons 1 vs. 1), while any comparison of different states (0 vs. 1 or 1 vs. 0) generates a value of 1.

### Ethical Approval

This study was approved by the Human Research Ethics Committee of the Vall d'Hebron University Hospital. All patients who agreed to participate in the study signed an informed consent form.

## Results

### Patient Characteristics

This study included 27 patients with chronic ChD, identified *via* positive serology (IgG anti-*T. cruzi*) and real-time PCR for ChD, who were clinically managed at the Infectious Disease Clinic of the Vall d'Hebron University Hospital, Barcelona, Spain between 2015 and 2019. All patients reside in Barcelona, Spain, and are immigrants from Bolivia, 8/27 (29.6%) being male and 19/27 (70.4%) female (Table 1). The mean age of the patients was 47.1 ± 12.44 years (Table 1). Taking into account clinical features, echocardiography (ECG) and radiological exams, 17/27 (62.9%) of the patients, presented the indeterminate form of ChD and 10/27 (37.1%) the cardiomyopathy form. All patients with Chagas cardiomyopathy' were categorized in stage B1 of clinical evolution according to the European Society of Cardiology (13). No patient had the digestive, nervous, or mixed clinical forms of ChD.

**Table 1:** General information of patients with Chagas disease involved in the study.

**Sample code**	**Age** **(years)**	**Gender**	**Clinical form**	**DTU**
1	32	F	Cardiac	TcV
2	27	F	Indeterminate	TcV
3	43	M	Cardiac	TcV
4	65	F	Cardiac	TcV
5	68	F	Cardiac	TcV
6	39	M	Indeterminate	TcV
7	60	F	Indeterminate	TcV
8	41	M	Indeterminate	TcV
9	46	M	Indeterminate	TcV
10	38	F	Cardiac	TcV
11	56	F	Indeterminate	TcV
12	67	F	Indeterminate	TcV
13	35	M	Indeterminate	TcV
14	44	F	Indeterminate	TcV
15	56	F	Indeterminate	TcV
16	28	M	Indeterminate	TcV
17	27	F	Indeterminate	TcV
18	39	F	Indeterminate	TcV
19	37	F	Indeterminate	TcV
20	56	F	Indeterminate	TcV
21	60	F	Cardiac	TcV
22	53	F	Cardiac	TcV
23	56	F	Indeterminate	TcV
24	44	M	Cardiac	TcV
25	62	M	Cardiac	TcV
26	43	F	Cardiac	TcV
27	50	F	Indeterminate	TcV

*F, Female; M, Male; TcV, T. cruzi DTU; DTU, Discrete Typing Unit*.

### Intra DTU TcV Genetic Variability of *Trypanosoma cruzi*

**Figure 1:**
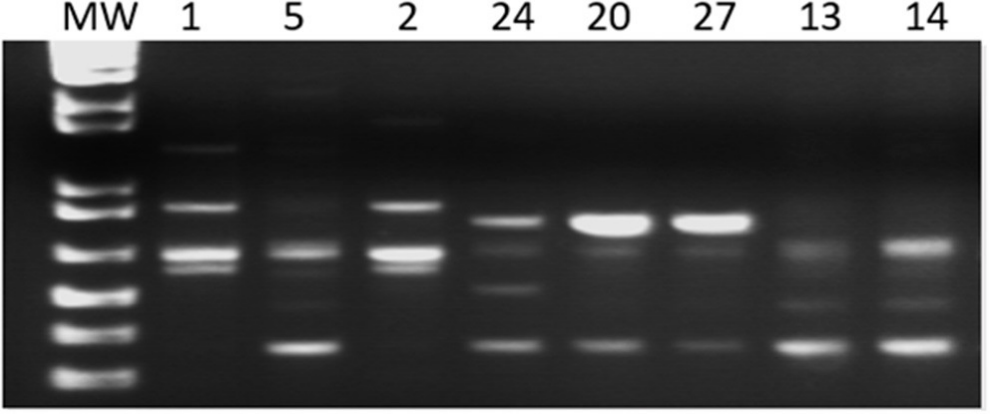
Gel representative of the amplified products of the LSSP-PCR. MW–Molecular Weight: 1 kb; 1, 5, 2, 24, 20, 27, 13, and 14 patient samples.

**Figure 2:**
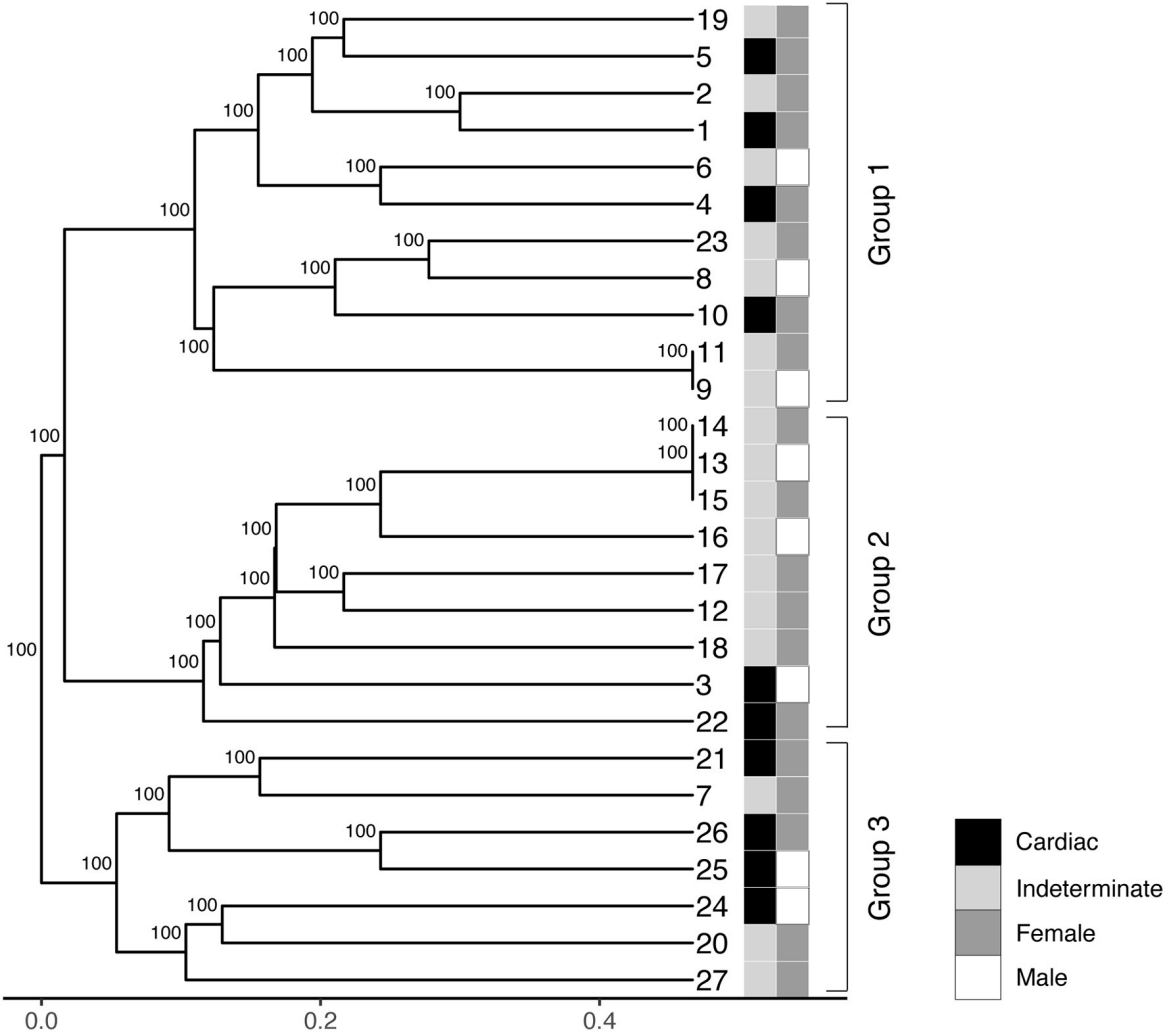
Phenogram containing UPGMA (unweighted pair-group method analysis) of the 27 samples of *T. cruzi* belonging to DTU TcV. The numbers on the horizontal scale were obtained from the similarity coefficient of data. Different colors represent the clinical forms (indeterminate and cardiac) and gender of each patient (male or female).

The LSSP-PCR analysis showed that the reaction amplified the 330 pb fragment of the *T. cruzi* kDNA in all 27 DNA samples from patients infected with TcV DTU. After the second amplification cycle, a total of 432 fragments were detected, and the products amplified were evaluated. The sizes of the bands varied between 80 and 1,500 bp (Figure 1). Sixteen profiles of the amplified products of the LSSP-PCR were obtained and only one (6.25%) was shared among all samples. The percentage of polymorphic loci was 93.84%, demonstrating the existence of a high rate of genetic variability among the samples. The cophenetic correlation coefficient, which verifies that the dendrogram preserved the distances in pairs between the original unmodeled data points, obtained a value of 0.851, confirming the high representation of the similarity matrices in the dendrogram. The UPGMA dendrogram distinguished *T. cruzi* DTU TcV samples into three distinct groups (Figure 2). Patients with the indeterminate form of ChD were clearly grouped in two groups (group 1 and 2) of the dendrogram, what showed that the genomes of these protozoa share certain alleles that may indicate common genetic characteristics and less intraspecific variability. For patients with the cardiac form of ChD, no sharing of distinct alleles was observed, indicating a higher rate of intraspecific genetic variability and complexity (Figure 2).

**Figure 3:**
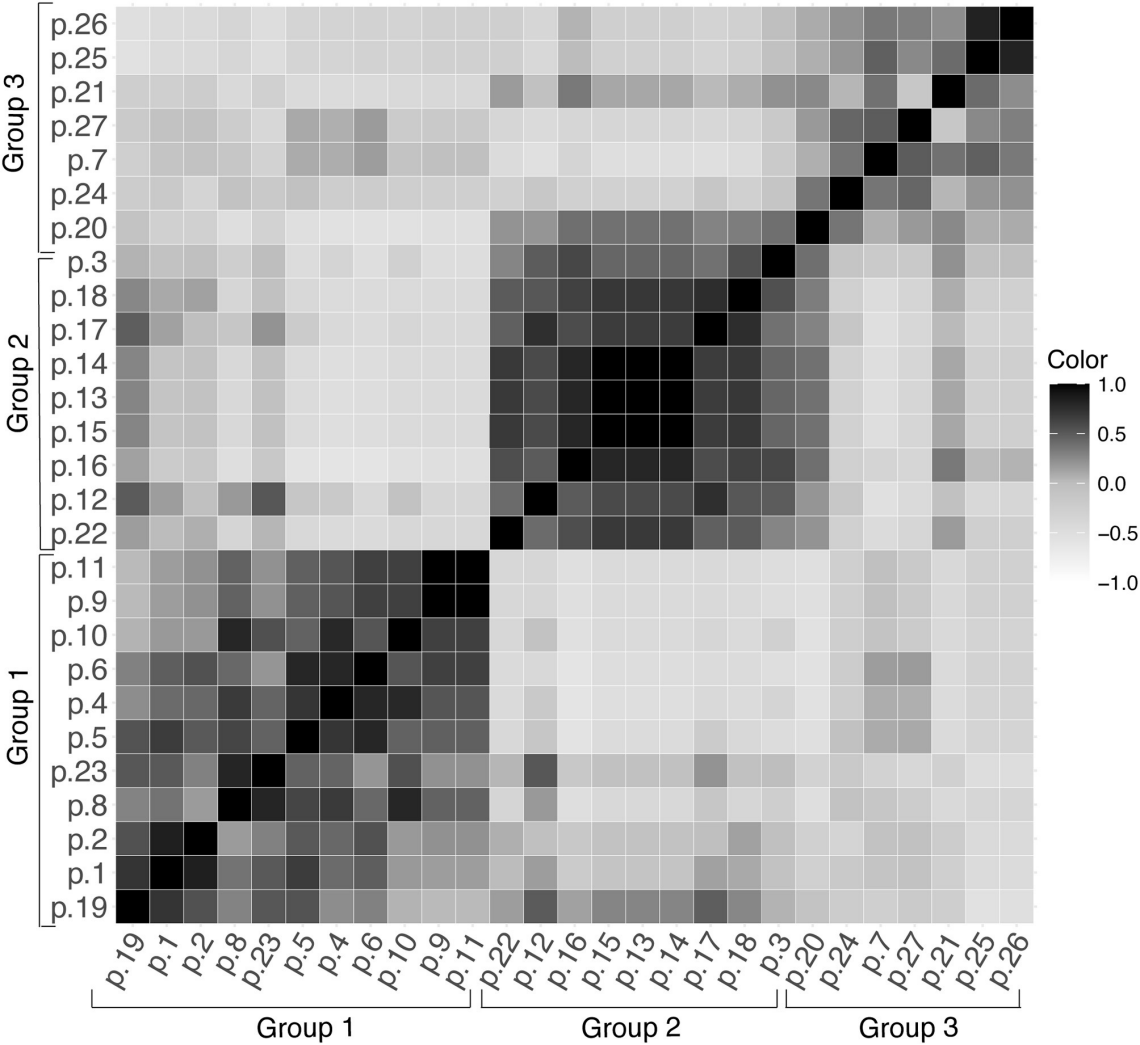
DICE similarity coefficient represented in the range of 0 to ± 1 for the 27 samples of *T. cruzi* (DTU TcV), (p, patient).

The DICE similarity coefficient corroborated the data contained in the dendrogram and UPGMA. This analysis was used to assess the variability of alleles in each sample in the range 0 to ± 1 (Figure 3). When comparing DNA samples from patients with the Indeterminate clinical form of Chagas disease, it was observed that the color intensity in Figure 3 tends to become darker, approaching the number 1 staining score. This may indicate a high degree of similarity between these samples.

**Figure 4:**
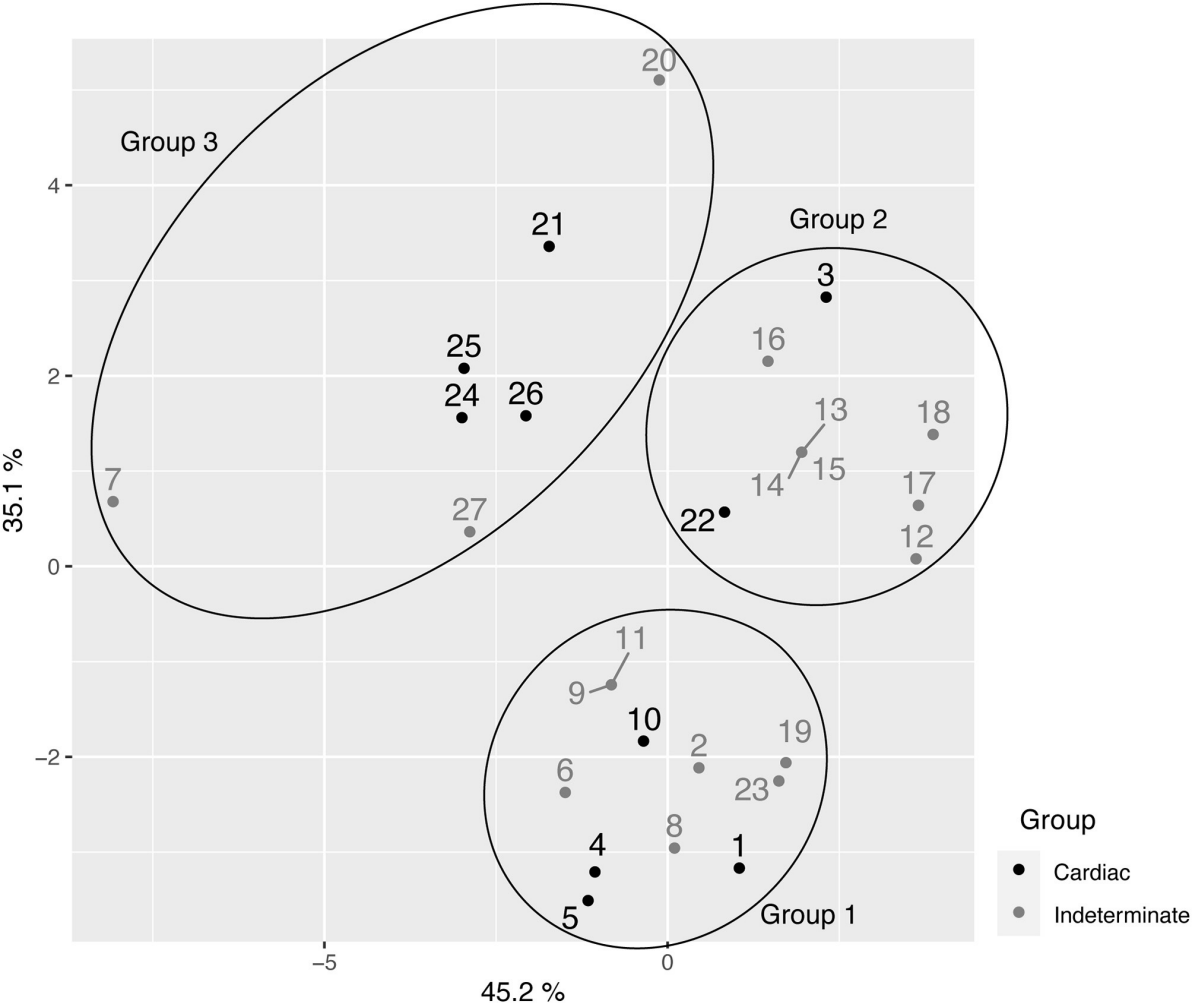
Principal coordinated analysis (PCoA) for the 27 samples of *T. cruzi* belonging to the TcV discreet typing unit (DTU).

The analysis of the principal coordinated analysis (PCoA) in the two axes demonstrated 80.30% (35.1 + 45.2%) of the variability between the components (Figure 4) and, in total, three distinct groups were formed, as shown in the UPGMA dendrogram. The average expected He was 0.296, indicative of moderate-high genetic diversity among the 27 samples. In addition, Shannon Weaver's diversity index (H′ = 3,159), a quantitative measure that defines the genetic diversity of species taking into account their variability, was also considered moderate to high. This assessment was used to describe the richness of the intra-DTU TcV variability of *T. cruzi*.

## Discussion

*Trypanosoma Cruzi* specie includes heterogeneous subpopulations that circulate in both domestic and wild cycles (22), and this diversity can be observed at the morphological (23), biological, antigenic (24), and genetic (5, 25) levels. Moreover, *T. cruzi* is currently subdivided into seven distinct genetic groups (DTUs TcI-TcVI and Tcbat) (4), and each DTU has its own characteristics (3). In order to better understand the disease in each geographical region it is necessary to study the molecular epidemiology of this parasite, inherently related to the main biological characteristics, which consequently have clinical implications on Chagas disease clinical features and evolution.

ChD is endemic in Latin America; however, the epidemiology of this infection has changed, mainly due to recent population migration for countries of distinct continents. At present, ChD is an important public health problem in other non-endemic regions, such as Europe (8). Immigration to Europe from Latin American countries has increased steadily over the last 2 years, especially to southern European countries, such as Spain and Italy. More recently, there is evidence for Latin American immigration to northern countries in Europe as well (10, 26). These population movements have increased the occurrence of ChD in these countries (9), since a considerable proportion of Latin American immigrants are chronically infected with *T. cruzi*, added to the occurrence of autochthone cases transmitted by mechanisms independent to triatomine vectors. Consequently, the number of reported cases of ChD with or without cardiac involvement has increased dramatically in recent years, especially in European countries, such as Spain, Italy, and Switzerland, where most Latino immigrants have settled (27, 28). Thus, studies aiming to evaluate the possible association between the intraspecific genetic variability of *T. cruzi* with the specific clinical forms of ChD are necessary.

It is known that DTU TcV is closely related to the domestic cycle of ChD associated with human disease in countries such as Argentina, Bolivia, Chile and Paraguay (6). This genotype is related to the presentation of clinical cardiac and digestive symptoms in patients from countries in the southern cone and little is known about their natural mammalian reservoirs (3). Previous studies have demonstrating the existence of genetic variability within TcI (29) and TcII (30) isolates. In the present study, a pioneering moderate-high rate of genetic variability within DTU TcV of *T. cruzi in a* specific population of Bolivian immigrants in Spain was assessed by LSSP-PCR. Furthermore, an association was detected between the indeterminate form of ChD and sharing certain alleles present in the parasite's genome.

It is relevant to emphasize that the LSSP-PCR methodology had been described and widely used to identify *T. cruzi* underlines before the first genotyping criteria emerged in 2009 (31–33). Burgos and collaborators used this methodology to evaluate the decrease of certain subclasses of the parasite kDNA minicircles in the peripheral blood and brain tissue of a patient infected with human immunodeficiency virus and with *T. cruzi* (TcII) during treatment for Chagas disease (34). Similarly, Costales and collaborators employed LSSP-PCR to investigate the presence of polyclonal infection of the parasite belonging to the TcI genotype in a cardiomyopathic patient with reactivation of the infection after heart transplantation (35).

Lages-Silva et al. (30) also tried to establish the association of intra-DTU genetic variability with the different clinical manifestations of patients infected with *T. cruzi*. It is worth mentioning that the authors used the same molecular technique (LSSP-PCR) employing different genes, and as in the present study, they were unable to detect an effective correlation, what demonstrated the need to search for genetic targets that are increasingly intrinsic in the parasite's genome. In the work involving DTU TcI (29), the study group sought to assess the association between the TcI genotype and the home and wild distribution of the genetic lineage in its hosts and biological aspects of the strains. It is worth mentioning that the TcV DTU is a hybrid strain, originating from several hybridization processes between TcI and TcII, with loss of heterozygosity between the progeny to produce TcIII and TcIV, followed by a second more recent hybridization event between TcII and TcIII to produce both, TcV and TcVI (36).

Lima and collaborators evaluated a family with two generations of patients with chronic ChD infected with DTU TcII, and attempted to establish the DTU/clinical forms correlation. However, no satisfactory results were obtained, possibly due to the low number of clinical samples. Despite of similarly to the present study, in addition, they reported low genetic variability/diversity in samples of the parasite belonging to the TcII genotype (37).

In our study the UPGMA analysis of the samples demonstrated the existence of three large groups within the *T. cruzi* TcV genotype, including a high rate of intraspecific genetic variability/diversity of this DTU. Similar data were obtained by some of us using *T. cruzi* DTU TcII and TcVI genotypes (38). We demonstrated, through UPGMA and PCoA of random amplification of polymorphic DNA, the existence of a high rate of genetic variability within DTU TcII and TcVI of *T. cruzi* in samples from patients with chronic ChD residing in an important endemic region of Minas Gerais state, Brazil.

Additionally, in accordance with the UPGMA results of our study, Macchiaverna and collaborators showed that the sequencing data of the *T. cruzi* TcMK (mevalonate kinase) gene in clinical samples from chronic patients in Argentina, revealed a low rate of genetic variability within the DTU TcV, and showing apparently two robust subgroups of isolates (39).

Few studies have undertaken a more detailed intra-DTU approach in an attempt to detect the appropriate correlations with the clinical forms of ChD presented by the patients. A more common approach has been to search correlation between *T. cruzi* DTUs and the clinical forms of ChD. However, it is reasonable to speculate that small genetic modifications or alterations in parasite genomes at intra-DTU level may be more related to the induction of distinct clinical manifestations of this disease, and even responses to a specific treatment against the *T. cruzi*, favoring a better understanding of the clinical and epidemiological aspects of this disease in endemic and non-endemic regions.

Therefore, knowing the genetic variability of *T. cruzi* intra-DTU TcV in immigrant patients with chronic ChD residing in Barcelona, Spain, is crucial to understanding the public health implications of ChD in European countries. Improving this understanding could contribute for the adequate design and planning of more effective public health interventions to improve the health of the immigrants and control the vertical transmission of ChD, which is a serious problem in Europe nowadays.

## Conclusions

This study demonstrated the existence of a moderate to high rate of intra-DTU TcV variability in *T. cruzi*, in a specific population of Bolivian immigrants in Spain. Being demonstrated association of sharing of certain alleles of the parasite with the absence of clinical manifestations in patients harboring the indeterminate clinical form of ChD, a trend to be assessed in a larger population. The information provided in this study could affect the planning of more effective public health interventions to improve the health of immigrants, vertical transmission control, and improvement of ChD treatment in countries with predominance of infection by TcV genotype. The results of this study support the need to search for increasingly intrinsic and specific targets in the genome of *T. cruzi* to be correlated with its main biological properties and clinical features in patients with chronic ChD.

## Data Availability Statement

The original contributions presented in the study are included in the article/supplementary material, further inquiries can be directed to the corresponding author/s.

## Ethics Statement

The studies involving human participants were reviewed and approved by Human Research Ethics Committee of the Vall d'Hebron University Hospital. The patients/participants provided their written informed consent to participate in this study.

## Author Contributions

MO, ES, AS, and IM: conceptualization. MO, ES, and IM: data curation. MO: formal analysis, validation, and writing—original draft. MO and JM-N: funding acquisition. MO, ML, and JS: investigation. MO, ES, and AS: methodology. JM-N and IM: project administration and supervision. MO, ES, ML, and IM: resources. MO, ES, AS, ML, JM-N, and IM: visualization. MO, MS, ML, JS, JM-N, and IM: writing—review and editing. All authors contributed to the article and approved the submitted version.

## Conflict of Interest

The authors declare that the research was conducted in the absence of any commercial or financial relationships that could be construed as a potential conflict of interest.
